# Research Trends in Periodontitis and Alzheimer's Disease: A Bibliometric Analysis Based on Web of Science and Scopus

**DOI:** 10.1016/j.identj.2025.109327

**Published:** 2025-12-17

**Authors:** Cuiting Chen, Qiongyu Chen, Han Zou, Chuanjiang Zhao, Xiaodong Wang

**Affiliations:** a Hospital of Stomatology, Sun Yat-Sen University, Guangzhou, Guangdong, China; b Department of Stomatology, The Third Affiliated Hospital, Sun Yat-Sen University, Guangzhou, Guangdong, China

**Keywords:** Alzheimer's Disease, Bibliometrics, CiteSpace, Periodontitis, VOSviewer

## Abstract

**Introduction and aims:**

This study aimed to conduct a comprehensive bibliometric analysis to identify global research trends, key contributors and emerging hot spots in the field investigating the association between periodontitis and Alzheimer's disease (AD).

**Methods:**

Scientific publications from 2002 to 2025 were retrieved from the Web of Science Core Collection (WoSCC) and Scopus databases. The data were analysed using VOSviewer, CiteSpace and the R package ‘bibliometrix’ to perform co-authorship, co-occurrence and citation analyses.

**Results:**

A total of 262 articles from WoSCC and 272 from Scopus were included in the analysis. China was the leading contributing country, and Shanghai Jiao Tong University was the most productive institution. The *Journal of Alzheimer's Disease* was identified as the most influential journal in this domain. Keyword co-occurrence analysis identified central research themes, including 'dementia', 'tooth loss', and '*Porphyromonas gingivalis*'. Citation burst analysis indicated that 'oral microbiome' and 'oral health' are currently emerging research frontiers.

**Conclusion:**

This is the first bibliometric study to systematically map the intellectual structure and evolution of research linking periodontitis and AD. The findings underscore the strengthening link between oral inflammatory conditions and neurodegeneration.

**Clinical relevance:**

The analysis highlights a shifting focus towards mechanisms such as the oral microbiome and systemic inflammation, pointing to promising directions for future research aimed at novel preventive strategies and therapeutic interventions for AD.

## Introduction

Alzheimer’s disease (AD), a progressive neurodegenerative disorder and the leading cause of dementia, is characterised by cognitive decline, memory loss and behavioural changes. As the disease advances, it results in a progressive loss of independence and severe impairment in daily activities.^1^, ^2^, ^3^ With an estimated global prevalence of 33.9 million cases, AD is projected to affect 3 times as many people within the next 40 years, largely because of ageing populations and other demographic changes.^4^ The disease imposes profound societal and economic burdens, straining healthcare systems, caregivers and societies worldwide, with annual costs exceeding hundreds of billions of dollars.^5^^,^^6^ Neuroinflammation is recognised as a central mechanism driving the onset and progression of AD.^7^

Periodontitis, a chronic inflammatory condition, can elicit a systemic inflammatory response, which may in turn promote the development of neuroinflammation.^8^ The systemic spread of oral pathogens and their toxins has been linked to a low-grade inflammatory response, which is associated with both burning mouth syndrome (BMS)^9^ and potential adverse effects on brain function.^10^ This mechanism has spurred growing interest in the potential bidirectional relationship between periodontitis and AD.^11^ For instance, Holmer et al. reported that poor oral health, especially periodontitis, is associated with an increased risk of mild cognitive impairment and AD.^12^ Further supporting this, Wu et al. demonstrated in a mouse model that the periodontal pathogen *Fusobacterium nucleatum* aggravates AD’s pathology through microglial activation, leading to enhanced beta-amyloid accumulation and cognitive decline.^13^ However, recent meta-analyses caution that although periodontitis appears linked to dementia and AD, the evidence remains inconclusive because of substantial heterogeneity across studies.^14^^,^^15^ Despite this complexity, several common mechanisms have emerged, including shared hub genes (e.g. *PDGFRB, VCAN, TIMP1, CHL1, EFEMP2, IGFBP5*) and immune infiltration profiles.^16^ Moreover, clinical markers of periodontitis correlate with neuroimaging features indicative of AD risk.^17^ Given that periodontitis is a modifiable risk factor, its association with AD merits significant public health attention. There is an urgent need for a comprehensive synthesis of current evidence to outline scientific progress and pinpoint priorities for future research.

Bibliometrics is a scientific method used for the quantitative analysis and evaluation of research. It is instrumental in elucidating research trends, identifying seminal studies and mapping the evolution of specific fields over time.^18^ Although several bibliometric studies have investigated periodontitis and AD using data from either the Web of Science Core Collection (WoSCC) or Scopus databases, reliance on a single database limits the reliability of their findings.^19^^,^^20^ Consequently, there remains a lack of bibliometric analyses specifically focused on the relationship between periodontitis and AD that incorporate multiple databases. To address this gap, the present study aims to identify developmental trends in research linking periodontitis and AD by performing a comprehensive bibliometric analysis of relevant literature retrieved from both WoSCC and Scopus databases.

## Materials and methods

### Search strategies and data collection

A literature search on the WoSCC and Scopus^21^ was performed to investigate periodontitis and AD. WoSCC is renowned for its meticulous indexing and citation analysis capabilities, providing curated content from high-impact journals. It allows for an accurate assessment of research influence, author productivity and institutional performance. Its long-standing history and strict inclusion criteria ensure high-quality and credible data, making it particularly valuable for tracking longitudinal research trends and citation dynamics in core scientific literature. Scopus, on the other hand, offers extensive multidisciplinary coverage, including peer-reviewed literature from science, technology, medicine, social sciences and arts and humanities. It is known for its comprehensive journal inclusion, international scope and advanced citation-tracking tools.^22^ Scopus also includes conference proceedings and book chapters, enabling a more inclusive view of emerging research. Its user-friendly analytics and visualisation features make it especially suitable for bibliometric and trend analyses across diverse academic domains.

The search query was formulated as (TS=(Periodontitis OR ‘Periodontal Inflammation’ OR ‘Chronic Periodontitis’ OR ‘Aggressive Periodontitis’ OR ‘Refractory Periodontitis’ OR ‘Advanced Periodontal Disease’)) AND TS=(Alzheimer* OR ‘Alzheimer disease’ OR ‘Alzheimer’s disease’ OR ‘Senile Dementia’).^23^, ^24^, ^25^, ^26^, ^27^ The inclusion criteria were (1) English language; (2) published between 1 January 2002 and 25 March 2025; and (3) articles related to periodontitis and AD meeting the search formula. Records such as letters, conference abstracts, conference proceedings, literature reviews and other non-article document types were excluded. To avoid discrepancies arising from database updates, the literature retrieval was conducted on 25 March 2025. Bibliographic information was exported using the ‘Full record and cited references’ and ‘plain text’ formats during the filtering process. Data were collected in text format, encompassing the number of publications and citations, titles, author information, institutions, countries/regions, keywords and journals for subsequent bibliometric analysis. The screening process was conducted by two independent reviewers. In cases of disagreement between reviewers, consensus was achieved through discussion. If necessary, a third reviewer was consulted for the final decision.

### Statistical analysis

We selected appropriate software to analyse different aspects of our research results for optimal presentation. In this research, we used the R-bibliometrix package (version 3.2.1, http://www.bibliometrix.org), CiteSpace (version 6.1. R2) and VOSviewer (version 1.6.20) for visual analysis of the selected literature. VOSviewer played a pivotal role in mapping collaborations among institutions and authors, as well as in analysing co-authorship, citation patterns and co-citation networks.^28^ In the present study, VOSviewer primarily conducted the following analyses: country and institution analysis, journal and co-cited journal analysis, author and co-cited author analysis and keyword co-occurrence analysis. In the maps generated by VOSviewer, each node represents an element, such as a country, institution, journal or author. The size and colour of each node indicated the quantity and classification of these elements while the thickness of the lines connecting the nodes illustrated the extent of collaboration or co-citation among them.

CiteSpace was used to identify keyword bursts. The parameters were configured as follows: time slicing was conducted from January 2002 to March 2025, with keywords designated as the node type. The threshold for keyword nodes was established at 5 for each fragment, and pruning was executed using the pathfinder networks method. The selection of parameter values was based on previous bibliometric studies and methodological standards in the field.^29^ For instance, the keyword node threshold in CiteSpace was set at 5 to ensure a balance between sensitivity and specificity, capturing meaningful trends while minimising noise. The rest was set to a specific value, and the cutting method could be set as necessary in accordance with the selected nodes, such that the result would be the most stable, transparent and intuitive. Visual analysis was performed based on these parameters to generate a timeline of keywords associated with periodontitis and AD. Besides, in terms of pruning method, compared with minimum spanning tree (MST), pathfinder used in this study offers completeness and yields only a unique solution to ensure the stability and repeatability of the results.^30^

The R package ‘bibliometrix’ was used to conduct a comprehensive bibliometric analysis. The H-index was employed to quantify the academic impact of both individuals and journals. This metric reflects a researcher's productivity as well as the citation impact of their publications, thereby providing a balanced measure of academic influence.^31^^,^^32^ To assess the prestige and citation influence of journals, we used the Journal Citation Reports (JCR) quartiles and the Impact Factor (IF). JCR quartiles categorise journals into 4 tiers, with Q1 representing the highest level of academic impact. The IF quantifies the average number of citations received by articles published in a journal over the preceding 2 years. For this analysis we employed the most recent 2024 release of JCR and IF data to ensure an up-to-date assessment of journal prestige and citation influence. Additionally, Microsoft Office Excel 2019 was used for the quantitative analysis of the publication data.

## Results

### An overview of publications

**Fig. 1:**
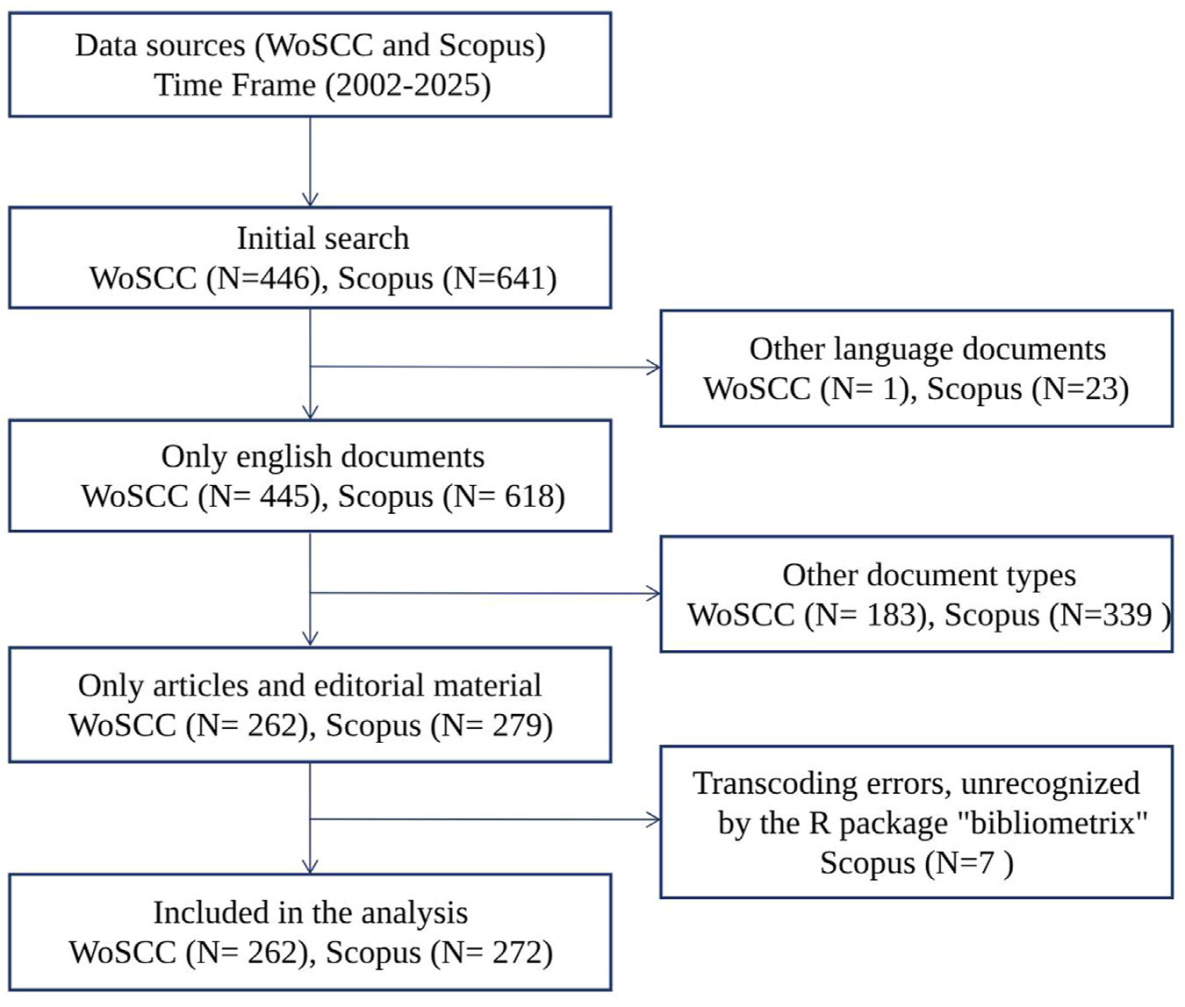
Flowchart of the literature search process.

The flowchart illustrating the data-screening process for WoSCC is shown in Figure 1 and Supplemental Figures S1 and S2. Initially, 446 publications were identified. After screening, 262 studies were included in the bibliometric analysis (Supplemental Figure S1). Our investigation revealed that 1,650 authors from 509 institutions across 51 countries contributed to the production of these 262 manuscripts in WoSCC. These works were published in 128 journals, citing a total of 9,914 references. The flowchart illustrating the data-screening process for Scopus is shown in Supplemental Figure S2. Initially, 641 publications were identified. After screening, 272 studies were included in the bibliometric analysis for Scopus (Supplemental Figure S2). Our investigation revealed that 1,550 authors contributed to the production of these 272 manuscripts for Scopus. These works were published in 164 journals and included a total of 664 author’s keywords. The references from WoSCC and Scopus are presented in Supplemental Tables S1 and S2, respectively.

**Fig. 2:**
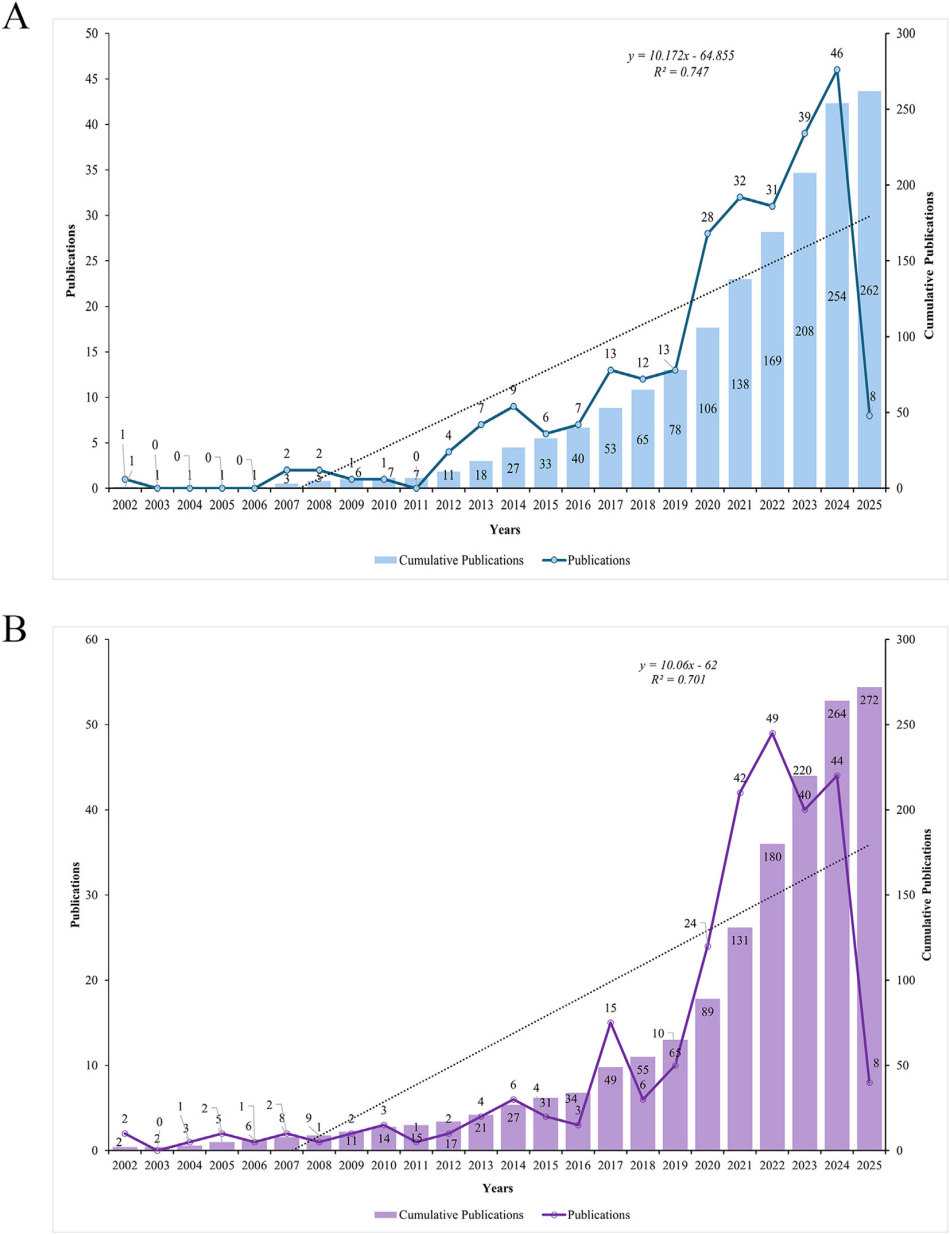
Annual publication trends on the role of periodontitis and Alzheimer's disease in WoSCC (A) and Scopus (B).

As shown in Figure 2A and 2B, the total number of publications concerning the role of periodontitis and AD showed significant growth over time with a similar trend between the two databases. The initial number of publications in 2002 was 1. From 2002 to 2010, the field experienced a gradual and relatively slow increase in the number of publications. By contrast, from 2011 to 2025, the field witnessed a surge in research output. The number of publications increased rapidly, especially between 2017 and 2023.

The most frequently cited article, titled ‘*Porphyromonas gingivalis* in Alzheimer’s disease brains: Evidence for disease causation and treatment with small-molecule inhibitors’, was published in 2019 in *Science Advances* and garnered a total of 1,198 citations.^33^ The article that ranked second in terms of citations, titled ‘Serum antibodies to periodontal pathogens are a risk factor for Alzheimer's disease’, was published back in 2012 in *Alzheimer's & Dementia*. It has garnered 327 citations.^34^ The third most cited article, ‘Inflammation and Alzheimer’s disease: possible role of periodontal diseases’, which appeared in the same journal in 2008, has also attracted considerable attention, amassing 282 citations.^35^

### Analysis of journals

A detailed overview of the top 20 most productive journals in the field of periodontitis and AD in WoSCC is shown in Supplemental Table S3. The *Journal of Alzheimer's Disease* stood out as the leading journal with an h-index of 12, an impact factor of 3.4 and 16 total publications (TP), ranking first in both TP and total citations (TC) (408), indicating its significant impact and prominence in this field. Other notable journals included the *Journal of Clinical Periodontology*, which ranked second in TP with 12 publications and an IF of 5.8, and *PLoS One*, which had 9 publications and an h-index of 8. As presented in Supplemental Table S4, based on Scopus, The *Journal of Alzheimer’s Disease* was also a prominent journal, with an h-index of 7, followed by *PLoS One* and *Frontiers in Aging Neuroscience*. While there are slight discrepancies between the two databases, both databases highlight the *Journal of Alzheimer's Disease* as the most influential contributor. Notably, the top 20 journals in Scopus included the *ACS OMEGA*, indicating more interest in inhibition of *Porphyromonas gingivalis* via advanced dental devices to avoid AD.^36^

**Fig. 3:**
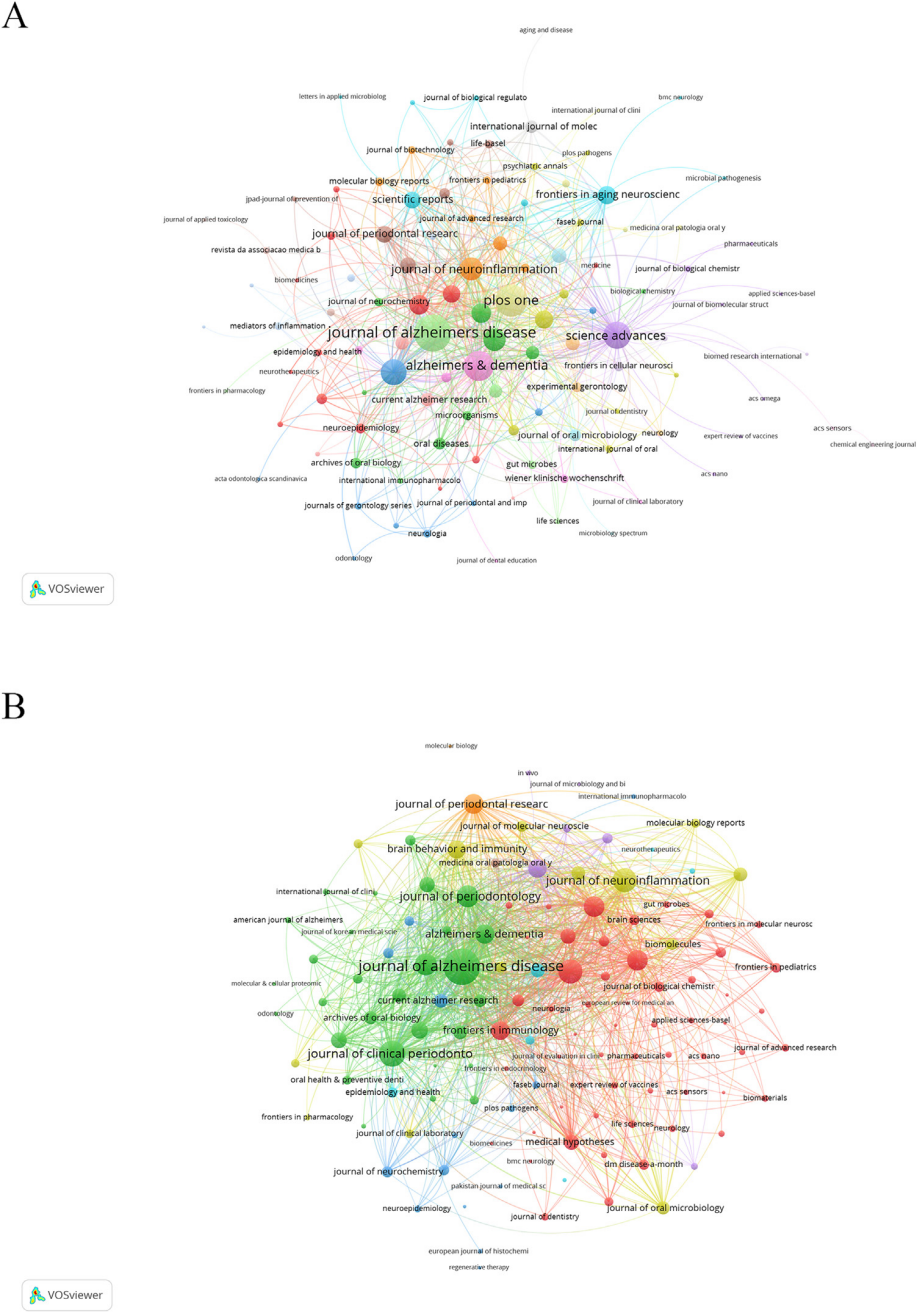
A, A journal co-occurrence network diagram. The frequency with which journals are cited together within the same articles reflects the thematic or topical connections between the research they publish. Colours indicate different research clusters. B, Journal bibliography coupling network diagram. The extent to which journals are linked is based on common references cited in their articles, indicating a shared intellectual foundation or research focus. Colours indicate different research clusters.

The journals featured in the research co-occurrence network diagram related to the field of periodontitis and AD are depicted in Figure 3A. In the analysis of the co-occurrence network, the 3 key journals with the highest total link strength were identified as the *Journal of Alzheimer's Disease* (185), *PLoS One* (143) and *Alzheimer's & Dementia* (124). The coupling network diagram of journals is presented in Figure 3B. In the analysis of the coupling network, the 3 key journals with the highest total link strength were identified as the *Journal of Alzheimer's Disease* (5,587), *Journal of Clinical Periodontology* (2,878) and *PLoS One* (2,485).

### Analysis of countries

**Fig. 4:**
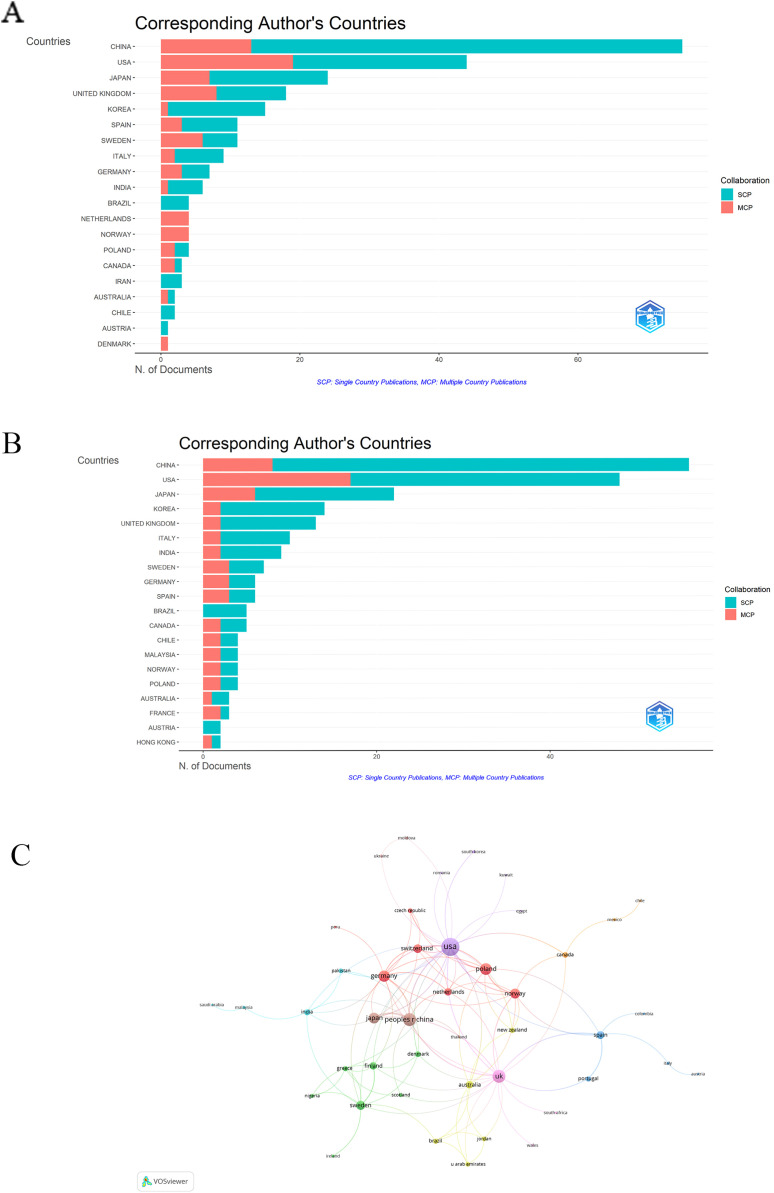
Distribution of corresponding author's publications by country in WoSCC (A) and Scopus (B). MCP, multiple country publications; SCP, single country publications. C, A visualisation map depicting the collaboration among different countries. Nodes indicate the publication count, and the thickness of links shows the strength of co-authorship collaborations. Colours indicate different research clusters.

Figure 4A illustrates the top 20 most prolific countries in terms of publications within this field in WoSCC. China emerged as the most productive nation, publishing 75 articles, followed by the United States with 44 articles and Japan with 24 articles (Figure 4A and Supplemental Table S5). The trend from the Scopus database mirrors that of WoSCC, where China emerged as the most prolific nation in terms of TP, with 56 articles, followed by the United States with 48 articles and Japan with 22 articles (Figure 4B and Supplemental Table S6).

The visualisation map depicting the collaboration among different countries in the field of periodontitis and AD is provided in Figure 4C. Among the 46 countries involved in international collaborations with a minimum of 1 article, the United States has the highest number of collaborations with other countries (57), followed by China (30) and the United Kingdom (30).

### Analysis of authors

The publication and citation profiles of high-impact authors in the field of periodontitis and AD research in WoSCC are presented in Supplemental Table S7. Wu Zhou emerged as a leading author with an h-index of 9, indicating a significant number of publications that have been cited at least 9 times each. He has published 9 papers since their first publication year in 2013, with a total of 514 citations, ranking them second in total citations. Ni Junjun follows closely with an h-index of 8 and has published 8 papers since 2013, which have accumulated 449 citations, ranking them third in total citations. This result was consistent with that from the Scopus database (Supplemental Table S8).

**Fig. 5:**
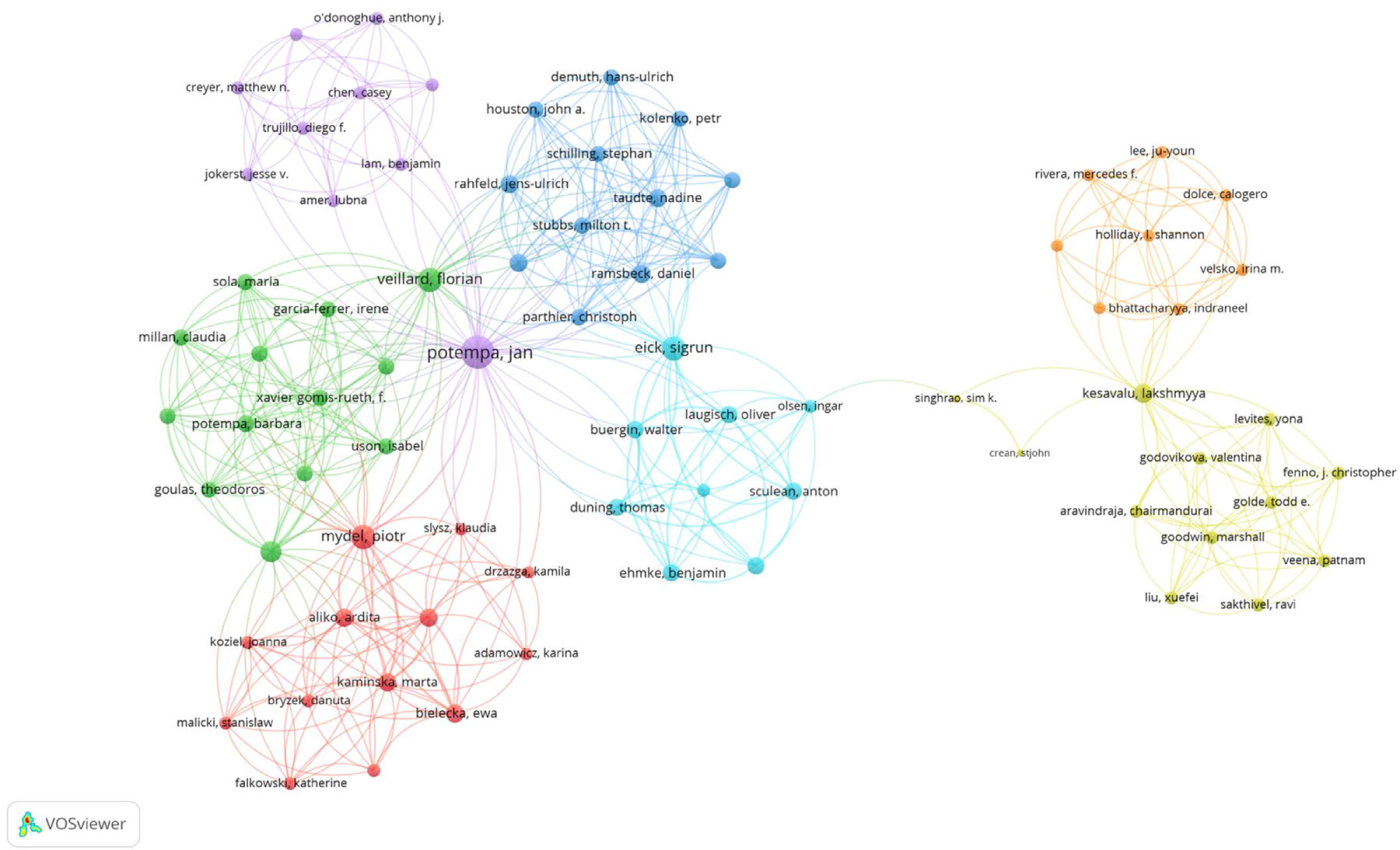
A visualisation map depicting the collaboration among different authors. Nodes represent authors, with size indicating the publication count. Links represent co-authorships, with thickness showing collaboration strength. Colours indicate different research clusters. Link strength in collaboration networks measures the frequency of co-authorship between authors, indicating the level of collaborative research.

The visualisation map depicting the collaboration among different authors is presented in Figure 5. Wu Zhou emerged as the most prominent author with the highest total link strength (57). Ni Junjun followed closely with a total link strength of 54. Other notable contributors include Song Zhongchen and Zhou Wei, both having a total link strength of 53.

### Analysis of institutions

**Fig. 6:**
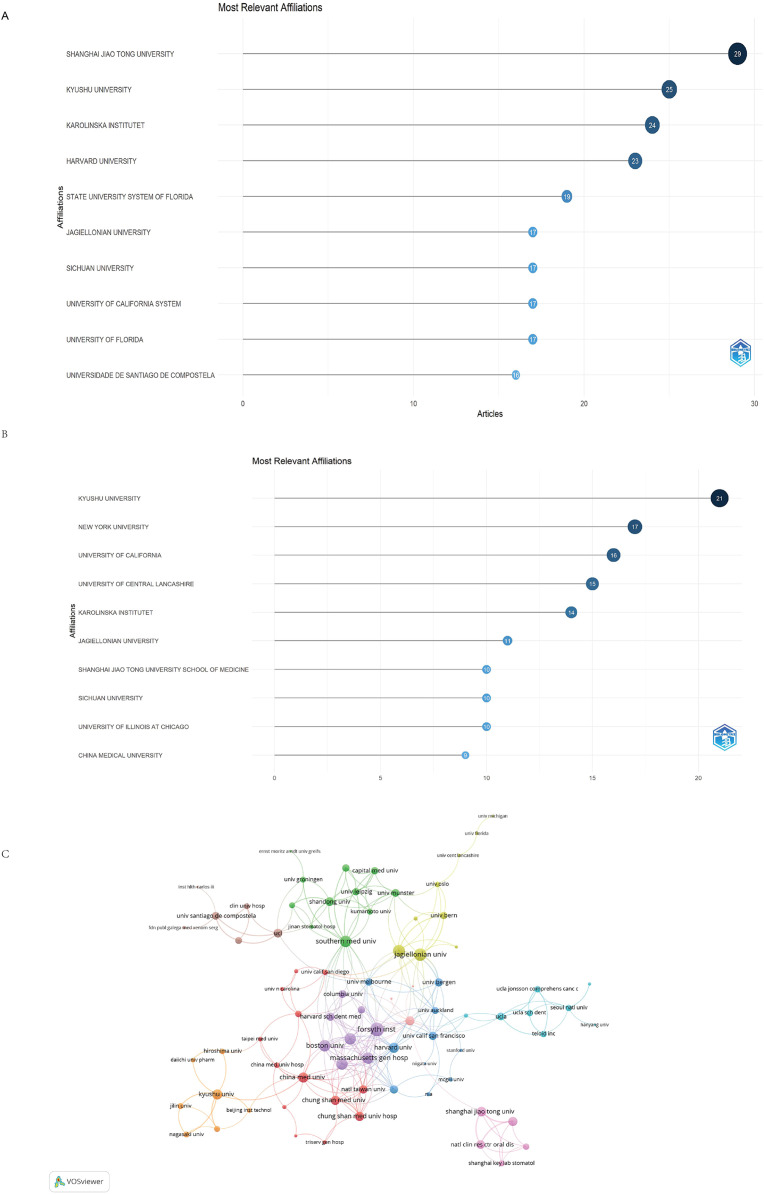
Top 10 institutions by article count and rank in WoSCC (A) and Scopus (B). The circle size shows the article count. Darker shades indicate higher ranks. C, A visualisation map based on WoSCC depicting the collaboration among different institutions. Nodes represent institutions, with size indicating the publication count. Links represent co-authorships, with thickness showing the collaboration strength of co-authorship collaborations. Colours indicate different research clusters.

Based on the WoSCC database, Shanghai Jiao Tong University led with the highest number of published articles, totalling 29, closely followed by Kyushu University (25) and Karolinska Institutet (24) (Figure 6A). By contrast, data from the Scopus database showed that Kyushu University was the most productive institution (21), followed by New York University (17) and the University of California (16) (Figure 6B).

The collaboration among different institutions is presented in Figure 6C. Forsyth Institute, with 6 documents and 1,362 citations, had the highest total link strength of 33, reflecting its central role in the global research network. Jagiellonian University, which published 11 documents and received 1,441 citations, had a total link strength of 26. The University of Louisville, with 9 documents and 1,402 citations, demonstrated strong collaboration with a total link strength of 25, indicating its influential position in the research community (Figure 6C).

### Analysis of keywords

**Fig. 7:**
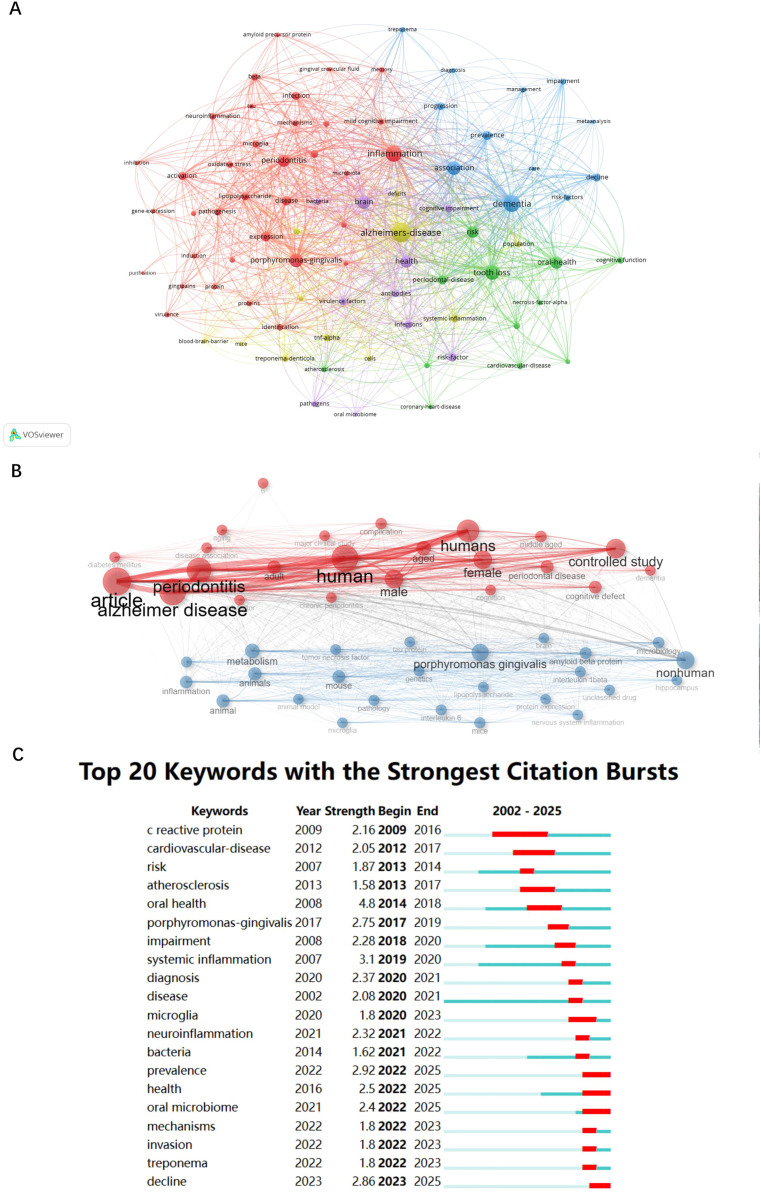
A, Visual analysis of keyword co-occurrence network analysis. Each node represents a keyword, with its size indicating its frequency of occurrence. Links between nodes represent co-occurrence in the same documents, with thicker lines showing stronger associations. Link strength measures the frequency of co-occurrence between keywords. Colours indicate different research clusters. B, Clustered keyword factorial analysis (Scopus; R bibliometrix). Each node represents a keyword, with its size indicating its frequency of occurrence. Links between nodes represent co-occurrence in the same documents, with thicker lines showing stronger associations. Link strength measures the frequency of co-occurrence between keywords. Colours indicate different research clusters. C, The top 20 keywords with the strongest citation bursts. The blue lines represent the period, and the red lines indicate the burst periods of the keywords.

In the keyword co-occurrence network in WoSCC, the first cluster centres on cognitive decline, in which ‘dementia’ (56 occurrences, total link strength of 245) highlights the focus on cognitive decline in relation to periodontitis. Another cluster emphasises oral health and microbial factors, with keywords such as ‘tooth loss’ (42 occurrences, total link strength of 195) and ‘*Porphyromonas gingivalis*’ (37 occurrences, total link strength of 167), highlighting the investigation into microbial factors and their role in systemic health, particularly the connection between oral pathogens and AD (Figure 7A and Supplemental Table S9). Based on the keyword co-occurrence analysis from the Scopus database, 2 main clusters were identified. The red cluster, representing clinical and population-based research, includes terms such as ‘human’, ‘periodontitis’, ‘female’, ‘aged’ and ‘cohort analysis’. The blue cluster, representing basic experimental and mechanistic studies, comprises keywords such as ‘*porphyromonas gingivalis*’, ‘mouse’, ‘inflammation’, ‘amyloid beta protein’ and ‘metabolism’ (Figure 7B and Supplemental Table S9).

### Analysis of burst keywords

The burst analysis of keywords from 2002 to 2025 was conducted to reveal the evolution of research trends in this field (Figure 7C; WoSCC). The keyword ‘oral health’ showed a significant citation burst from 2014 to 2018, indicating heightened research interest during this period. Similarly, ‘systemic inflammation’ had a notable burst from 2019 to 2020, reflecting active research and significant contributions to the literature. In addition, during the years leading up to 2025, the keywords ‘bacteria’ and ‘oral microbiome’ frequently emerged in publications, indicative of their being the research hot spots in the fields of periodontitis and AD during that period.

## Discussion

### General information

This bibliometric analysis of periodontitis and AD research highlighted the dynamic growth and evolving trends within this field from 2002 to 2025. Keyword co-occurrence analysis revealed that research topics covered dementia, tooth loss and *Porphyromonas gingivalis*. Keyword analysis revealed that recent research has increasingly focused on ‘oral microbiome’ and ‘oral health’, indicating emerging areas of interest in this field.

The United States' leading role in this research area may also be influenced by the high prevalence of AD in this country. The ageing population, lifestyle factors and access to healthcare services contribute to the need for extensive research on these conditions. Higher incidence rates drive both the demand for research and the funding available to address these public health challenges.^37^ Furthermore, the growing incidence of AD and periodontitis in China might be motivating institutions like Shanghai Jiao Tong University to focus on this research, aiming to address the public health implications and develop effective interventions.^38^ Authors such as Wu Zhou have made significant academic contributions, which may be attributed to their expertise, access to cutting-edge research facilities and strong collaborative networks. One of Wu Zhou’s studies found that chronic systemic exposure to *Porphyromonas gingivalis* lipopolysaccharide, associated with periodontitis, promotes tau hyperphosphorylation and neuroinflammation via GSK3β activation in a mouse model of AD, leading to cognitive deficits. This suggests that periodontitis may exacerbate Alzheimer's pathology, highlighting GSK3β as a potential therapeutic target to mitigate the disease's progression linked to oral health.^39^ This study has had a notable impact on bibliometric trends, spurring subsequent research focusing on the connection between oral pathogens and neurodegenerative diseases, contributing to the growing body of literature in this area.

### Research hot spots

Keyword co-occurrence analysis of periodontitis and AD reveals two dominant research clusters, one centred on cognitive decline and neurodegenerative diseases and the other on microbial factors linked to oral health. High-frequency terms such as ‘dementia’ reflect a research emphasis on how periodontitis may contribute to neurodegeneration,^33^ whereas such keywords as ‘tooth loss’ and ‘*Porphyromonas gingivalis*’ underscore the role of specific oral pathogens in AD-related pathways.^33^ The association between periodontitis and AD is multifactorial, involving systemic inflammation and direct pathogen effects. Periodontitis elevates circulating pro-inflammatory cytokines (e.g. TNF-α, IL-1β, IL-6), which can cross the blood–brain barrier, activate microglia and promote neuroinflammation—a process implicated in amyloid-beta accumulation and tau pathology.^11^^,^^40^ Notably, *Porphyromonas gingivalis* has been detected in AD brain tissue. It can breach the blood–brain barrier via gingipain proteases, induce neuroinflammation and promote amyloid deposition.^33^ The bacterium may also travel along the glymphatic system, acting as a ‘Trojan horse’ to infiltrate the brain.^41^

Keywords such as ‘human’, ‘aged’ and ‘cohort analysis’ indicate a growing focus on clinical and epidemiological validation. A prospective study by Kubra et al. suggested that periodontitis may accelerate AD progression.^42^ Data from the PerioMind Colombia Cohort revealed that older adults with mild cognitive impairment (MCI) showed significantly worse periodontal health—particularly in gingival erythema and pocket depth—which correlated strongly with MCI risk.^43^ Although interventional studies remain limited, emerging evidence supports a protective role for periodontal treatment. One 12-year cohort study reported that older adults with periodontitis who received gingival treatment had a 38% lower risk of dementia and slower cognitive decline compared to untreated individuals.^44^ Another study linked periodontal conditions to AD-related neuroimaging markers, reinforcing a potential neurobiological connection.^17^

### Emerging topics

Keywords such as ‘impairment’ and ‘systemic inflammation’ experienced bursts between 2018 and 2020. The research during this phase likely centred on elucidating the pathways through which chronic inflammation, triggered by periodontal disease, could exacerbate or contribute to the development of AD. For instance, Qian et al. demonstrated that periodontitis exacerbates cognitive impairment in a mouse model of AD by increasing neuroinflammation and beta-amyloid levels. The findings suggest that periodontitis may worsen the pathological features of AD, thereby accelerating cognitive decline,^45^ which can also be found in elderly patients, which also suggested that improving periodontal health could be an important strategy for preventing or mitigating cognitive decline in at-risk populations.^46^

From 2020 to 2025, there has been a notable shift towards more specific and emerging areas of research, as indicated by bursts in keywords such as ‘oral microbiome’ and ‘bacteria’, reflecting the growing interest in understanding how the composition and behaviour of the oral microbiome may contribute to the pathogenesis of AD, possibly through mechanisms involving chronic inflammation, bacterial translocation or immune system modulation. Substantial research has explored the gut microbiome’s role in neuropathogenesis, but the association between the oral microbiome and AD remains comparatively understudied. Na et al. revealed that patients with AD who also have periodontitis exhibit a distinctive subgingival microbiome compared to cognitively unimpaired individuals with periodontitis.^47^ Guo et al. analysed the oral microbiome of AD patients and found that *Porphyromonas gingivalis* was predominant in the periodontal microbiome.^48^ However, another study showed different results that periodontitis patients with AD exhibited a higher prevalence of *Atopobium rimae, Dialister pneumosintes, Olsenella* sp. *HMT807, Saccharibacteria (TM7)* sp. *HMT348* and multiple *Prevotella* species compared to cognitively unimpaired periodontitis patients,^47^ with no significant differences in *Porphyromonas gingivalis* between the 2 groups. If a definitive causative link between the oral microbiome and AD is established, quantifying specific microbial abundances in the oral cavity could serve as a valuable biomarker for early AD risk or progression prediction. Besides, Nisin, a probiotic bacteriocin, mitigates brain microbiome dysbiosis, resulting in amelioration of AD-like pathology and neuroinflammation, thereby positioning itself as a promising therapeutic candidate for periodontitis-associated AD.^49^ Consequently, targeted modulation of the oral microbiota as an emerging frontier may represent a promising strategy for delaying or preventing the onset of AD.

### Comparison with previous bibliometric analyses

To our knowledge, the previous bibliometric studies have explored the relationship between periodontitis and AD. Juliana et al. performed a bibliometric analysis using only the Scopus database, offering a preliminary quantitative overview of publication trends and country distributions.^20^ Similarly, Han et al. relied exclusively on the WoSCC.^19^ The use of a single database in each case may have led to the omission of relevant literature from other sources, thereby introducing potential bias. In contrast, our study integrates both Scopus and WoSCC to enable cross-validation and enhance the reliability of the findings. Moreover, Han et al.’s analysis primarily focused on neurodegenerative diseases as a broader research domain rather than specifically addressing periodontitis and AD, which makes its thematic scope not fully overlap with our research topic. A third study combined bibliometric and systematic review methodologies to elucidate key pathways linking periodontitis to neuroinflammation. While valuable, that study did not project future research directions, thus offering limited guidance for subsequent investigations.^50^ Notably, all 3 prior studies identified the mechanistic links between periodontitis and AD as a current research hotspot—a conclusion corroborated by our analysis. Going beyond existing work, our study further suggests that shifts in the oral microbiota may represent promising predictive biomarkers and therapeutic targets, highlighting an emerging frontier with considerable potential to guide future research and clinical strategies.

## Strengths and limitations

The study presents several notable strengths. First, the comprehensive bibliometric analysis provides a detailed overview of research trends and key contributors in the field of periodontitis and AD, effectively mapping the landscape of this research area. Second, the use of multiple bibliometric tools facilitates robust visualisations of collaborations, keyword co-occurrences and emerging trends, thereby ensuring a well-rounded understanding of the data.

However, this study also encounters several limitations that warrant acknowledgement. The bibliometric analysis was conducted using the WoSCC and Scopus databases, which may have introduced linguistic and database bias. As a result, relevant studies published in languages other than English or indexed in other bibliographic platforms may have been overlooked, potentially underrepresenting contributions from non-English–speaking countries, particularly since China was the leading contributor. However, WoSCC and Scopus constitute comprehensive databases renowned for their reliable citation tracking and robust bibliometric data. Supplementary databases may provide ancillary insights, but WOSCC and Scopus deliver sufficiently representative coverage of research trends in this domain. Additionally, the exclusive reliance on high-frequency keywords fails to capture the full spectrum of scientific progress, as it may marginalise niche yet significant fields of study if self-citations are not accounted for.

## Conclusion

This study used bibliometric analysis to examine the literature pertaining to periodontitis and AD, with the objective of evaluating research trends and identifying prominent contributors in the field. The analysis uncovered significant research hotspots that underscore the relationship between oral health and neurodegenerative diseases, specifically highlighting the role of the oral microbiome in these conditions. Overall, this study provides a comprehensive overview of the field and offers valuable insights for researchers investigating the intersection of these 2 areas. The key findings indicate potential clinical implications, particularly concerning the influence of oral health on the progression of AD.

## Authors' contributions

*Conception and design:* Wang Zhao

*Administrative support:* Q. Chen

*Data analysis and interpretation:* C. Chen, Zou

*Writing—original draft:* C. Chen

*Writing—revision and editing:* All authors

## Funding

NA

## Declaration of competing interest

None declared

## Appendix Supplementary materials

**Fig. S1** Flowchart of the literature screening process in WoSCC. Image, application 1

**Fig. S2** Flowchart of the literature screening process in Scopus. Image, application 2

**Table S1** Details of the 262 articles selected for final analysis from the WoSCC database. Image, application 3

**Table S2** Details of the 272 articles selected for final analysis from the Scopus database. Image, application 4

**Table S3** Top 20 most productive journals in the field of periodontitis and Alzheimer's disease in WoSCC. **Table S4** Top 20 most productive journals in the field of periodontitis and Alzheimer's disease in Scopus. **Table S5** Publication and citation profiles of leading countries in WoSCC. **Table S6** Publication and citation profiles of leading countries in Scopus. **Table S7** Publication and citation profiles of high-impact authors in WoSCC. **Table S8** Publication and citation profiles of high-impact authors in Scopus. **Table S9** Keyword occurrences analysis. Image, application 5

## Footnotes

Supplementary material associated with this article can be found in the online version at doi:10.1016/j.identj.2025.109327.
