# Supplementary figures and images for: Research Trends in Periodontitis and Alzheimer's Disease: A Bibliometric Analysis Based on Web of Science and Scopus
Source: Int Dent J. 2025 Dec 17;76(1):109327. doi: 10.1016/j.identj.2025.109327 (PMC12828214; doi:10.1016/j.identj.2025.109327)

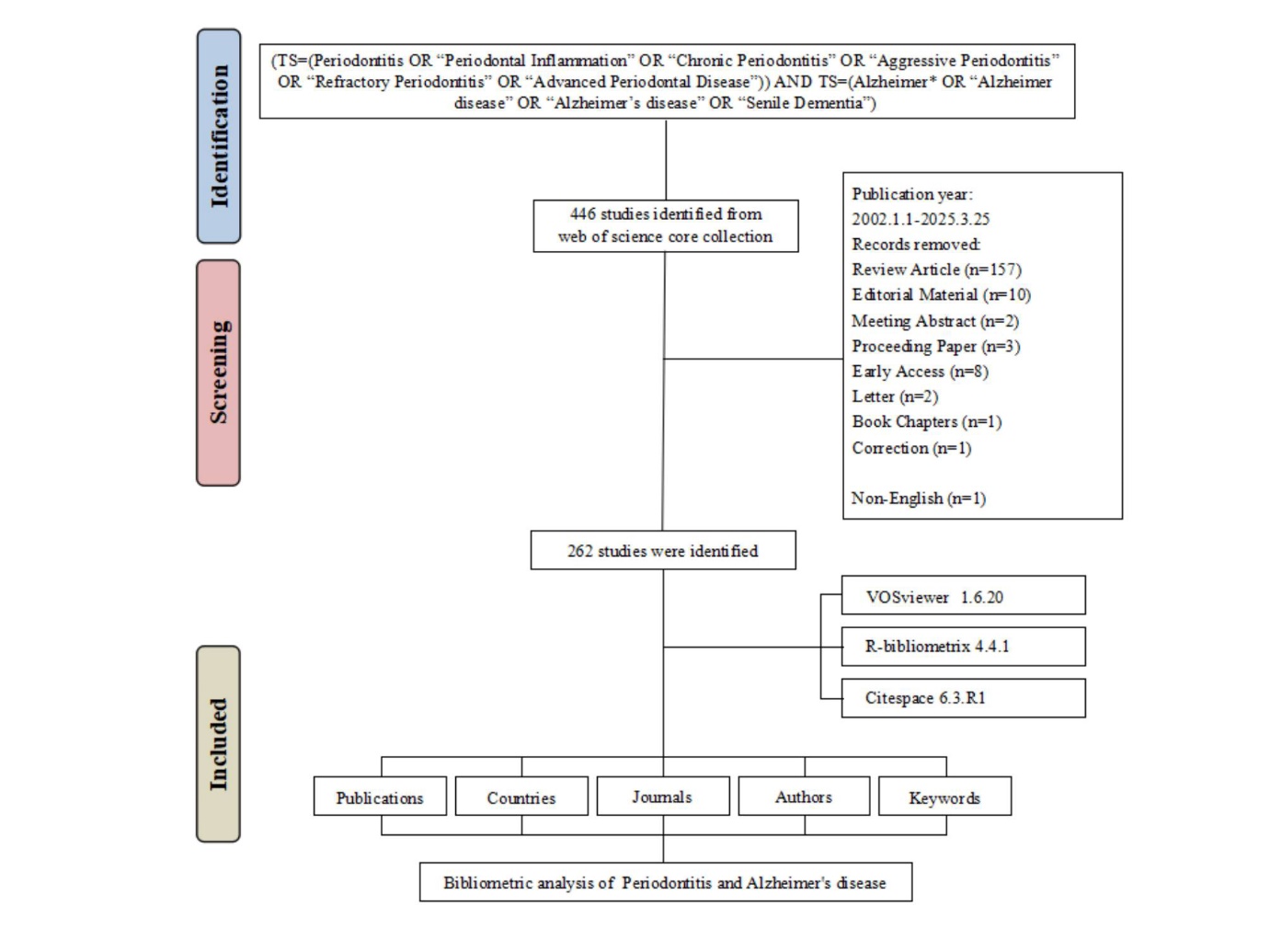

Supplement: Supplementary file 1 — Fig. S1 Flowchart of the literature screening process in WoSCC. [file mmc1.docx]

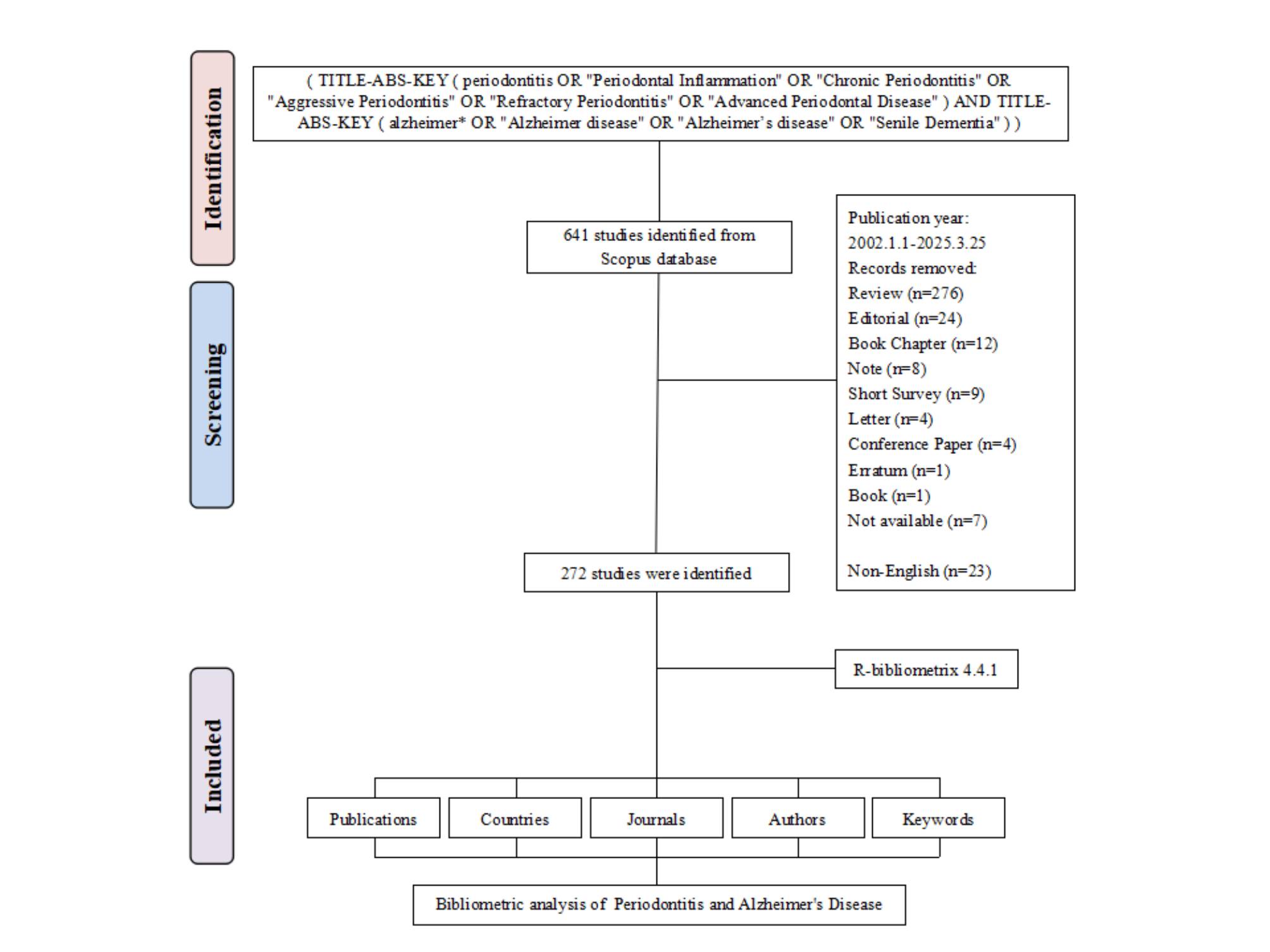

Supplement: Supplementary file 2 — Fig. S2 Flowchart of the literature screening process in Scopus. [file mmc2.docx]
