# Supplementary material for: Research Trends in Periodontitis and Alzheimer's Disease: A Bibliometric Analysis Based on Web of Science and Scopus
Source: Int Dent J. 2025 Dec 17;76(1):109327. doi: 10.1016/j.identj.2025.109327 (PMC12828214; doi:10.1016/j.identj.2025.109327)
Supplement: Supplementary file 5 — Table S3 Top 20 most productive journals in the field of periodontitis and Alzheimer's disease in WoSCC. Table S4 Top 20 most productive journals in the field of periodontitis and Alzheimer's disease in Scopus. Table S5 Publication and citation profiles of leading countries in WoSCC. Table S6 Publication and citation profiles of leading countries in Scopus. Table S7 Publication and citation profiles of high-impact authors in WoSCC. Table S8 Publication and citation profiles of high-impact authors in Scopus. Table S9 Keyword occurrences analysis. [file mmc5.docx]

**Table S3**. **Top 20 most productive journals in the field of periodontitis and Alzheimer's disease in WoSCC**

| **Journal** | **H_index** | **G_index** | **M_index** | **IF 2023** | **JCR 2023** | **TP** | **TP_rank** | **TC** | **TC_rank** | **PY_start** |
| --- | --- | --- | --- | --- | --- | --- | --- | --- | --- | --- |
| JOURNAL OF ALZHEIMERS DISEASE | 12 | 16 | 0.857 | 3.4 | 2 | 16 | 1 | 463 | 1 | 2012 |
| JOURNAL OF CLINICAL PERIODONTOLOGY | 9 | 12 | 0.375 | 5.8 | 1 | 12 | 2 | 445 | 2 | 2002 |
| PLOS ONE | 8 | 9 | 0.667 | 2.9 | 1 | 9 | 4 | 333 | 4 | 2014 |
| FRONTIERS IN IMMUNOLOGY | 6 | 6 | 1 | 5.7 | 1 | 6 | 9 | 81 | 26 | 2020 |
| JOURNAL OF PERIODONTOLOGY | 6 | 7 | 0.545 | 4.2 | 1 | 7 | 8 | 433 | 3 | 2015 |
| FRONTIERS IN AGING NEUROSCIENCE | 5 | 8 | 0.5 | 4.1 | 2 | 8 | 5 | 111 | 19 | 2016 |
| INTERNATIONAL JOURNAL OF MOLECULAR SCIENCES | 5 | 10 | 0.714 | 4.9 | 1 | 11 | 3 | 109 | 20 | 2019 |
| JOURNAL OF NEUROINFLAMMATION | 5 | 7 | 0.625 | 9.3 | 1 | 7 | 6 | 132 | 15 | 2018 |
| ALZHEIMERS & DEMENTIA | 4 | 5 | 0.222 | 13.1 | 1 | 5 | 10 | 269 | 5 | 2008 |
| EXPERIMENTAL GERONTOLOGY | 4 | 4 | 0.571 | 3.3 | 2 | 4 | 14 | 21 | 109 | 2019 |
| JOURNAL OF PERIODONTAL RESEARCH | 4 | 7 | 0.4 | 3.4 | 1 | 7 | 7 | 174 | 9 | 2016 |
| JOURNAL OF THE AMERICAN GERIATRICS SOCIETY | 4 | 4 | 0.308 | 4.3 | 1 | 4 | 17 | 187 | 8 | 2013 |
| SCIENTIFIC REPORTS | 4 | 5 | 0.364 | 3.8 | 1 | 5 | 11 | 159 | 11 | 2015 |
| ARCHIVES OF ORAL BIOLOGY | 3 | 3 | 0.25 | 2.2 | 2 | 3 | 20 | 57 | 36 | 2014 |
| BRAIN BEHAVIOR AND IMMUNITY | 3 | 3 | 0.333 | 8.8 | 1 | 3 | 21 | 88 | 22 | 2017 |
| CLINICAL ORAL INVESTIGATIONS | 3 | 4 | 0.375 | 3.1 | 1 | 4 | 13 | 53 | 39 | 2018 |
| FRONTIERS IN CELLULAR AND INFECTION MICROBIOLOGY | 3 | 4 | 0.6 | 4.6 | 1 | 4 | 15 | 88 | 23 | 2021 |
| GERODONTOLOGY | 3 | 3 | 0.231 | 2 | 2 | 3 | 23 | 116 | 18 | 2013 |
| MEDICAL HYPOTHESES | 3 | 4 | 0.5 | 2.1 | 3 | 4 | 18 | 10 | 243 | 2020 |
| ORAL DISEASES | 3 | 4 | 1 | 2.9 | 1 | 4 | 19 | 52 | 41 | 2023 |

Note(s): H_index: The h-index of the journal, which measures both the productivity and citation impact of the publications. IF: Impact Factor, indicating the average number of citations to recent articles published in the journal. JCR_Quartile: The quartile ranking of the journal in the Journal Citation Reports, indicating the journal's ranking relative to others in the same field (Q1: top 25%, Q2: 25%-50%, Q3: 50%-75%, Q4: bottom 25%). TP: Total Publications. TP_rank: Rank of Total Publications. TC: Total Citations. TC_rank: Rank of Total Citations. Average Citations: The average number of citations per publication. PY_start: Publication Year Start, indicating the year the journal started publication.

**Table S4**. **Top 20 most productive journals in the field of periodontitis and Alzheimer's disease in Scopus**

| **Journal** | **H_index** | **G_index** | **M_index** | **IF 2023** | **JCR 2023** | **TP** | **TP_rank** | **TC** | **TC_rank** | **PY_start** |
| --- | --- | --- | --- | --- | --- | --- | --- | --- | --- | --- |
| JOURNAL OF ALZHEIMER'S DISEASE | 7 | 10 | 0.583 | 3.4 | 2 | 10 | 1 | 335 | 3 | 2014 |
| PLOS ONE | 7 | 7 | 0.583 | 2.9 | 1 | 7 | 3 | 802 | 1 | 2014 |
| FRONTIERS IN AGING NEUROSCIENCE | 6 | 8 | 0.6 | 4.1 | 2 | 8 | 2 | 274 | 4 | 2016 |
| FRONTIERS IN IMMUNOLOGY | 6 | 6 | 1 | 5.7 | 1 | 6 | 5 | 170 | 7 | 2020 |
| INTERNATIONAL JOURNAL OF MOLECULAR SCIENCES | 5 | 7 | 1 | 4.9 | 1 | 7 | 3 | 60 | 15 | 2021 |
| JOURNAL OF CLINICAL PERIODONTOLOGY | 5 | 6 | 0.208 | 5.8 | 1 | 6 | 5 | 167 | 8 | 2002 |
| ALZHEIMER'S AND DEMENTIA | 4 | 5 | 0.222 | 13.1 | 1 | 5 | 7 | 728 | 2 | 2008 |
| JOURNAL OF DENTAL RESEARCH | 4 | 5 | 1 | 5.7 | 1 | 5 | 7 | 128 | 10 | 2022 |
| JOURNAL OF NEUROINFLAMMATION | 4 | 4 | 1.333 | 9.3 | 1 | 4 | 9 | 78 | 14 | 2023 |
| EXPERIMENTAL GERONTOLOGY | 3 | 3 | 0.429 | 3.3 | 2 | 3 | 14 | 85 | 12 | 2019 |
| FRONTIERS IN CELLULAR AND INFECTION MICROBIOLOGY | 3 | 4 | 0.6 | 4.6 | 1 | 4 | 9 | 125 | 11 | 2021 |
| GERODONTOLOGY | 3 | 4 | 0.231 | 2 | 2 | 4 | 9 | 56 | 16 | 2013 |
| JOURNAL OF ALZHEIMER'S DISEASE REPORTS | 3 | 3 | 0.6 | 2.8 | 2 | 3 | 14 | 20 | 20 | 2021 |
| MEDICAL HYPOTHESES | 3 | 4 | 0.333 | 2.1 | 3 | 4 | 9 | 42 | 18 | 2017 |
| SCIENTIFIC REPORTS | 3 | 4 | 0.333 | 3.8 | 1 | 4 | 9 | 130 | 9 | 2017 |
| ACS OMEGA | 2 | 2 | 0.333 | 3.7 | 2 | 2 | 16 | 52 | 17 | 2020 |
| ALZHEIMER'S RESEARCH AND THERAPY | 2 | 2 | 0.222 | 8 | 1 | 2 | 16 | 221 | 5 | 2017 |
| AMERICAN JOURNAL OF ALZHEIMER'S DISEASE AND OTHER DEMENTIAS | 2 | 2 | 0.167 | 2.7 | 2 | 2 | 16 | 84 | 13 | 2014 |
| ARCHIVES OF ORAL BIOLOGY | 2 | 2 | 0.167 | 2.2 | 2 | 2 | 16 | 38 | 19 | 2014 |
| BRAIN, BEHAVIOR, AND IMMUNITY | 2 | 2 | 0.222 | 8.8 | 1 | 2 | 16 | 206 | 6 | 2017 |

Note(s): H_index: The h-index of the journal, which measures both the productivity and citation impact of the publications. IF: Impact Factor, indicating the average number of citations to recent articles published in the journal. JCR_Quartile: The quartile ranking of the journal in the Journal Citation Reports, indicating the journal's ranking relative to others in the same field (Q1: top 25%, Q2: 25%-50%, Q3: 50%-75%, Q4: bottom 25%). TP: Total Publications. TP_rank: Rank of Total Publications. TC: Total Citations. TC_rank: Rank of Total Citations. Average Citations: The average number of citations per publication. PY_start: Publication Year Start, indicating the year the journal started publication.

**Table S5.** **Publication and citation profiles of leading countries in WoSCC**

| **Country** | **Articles** | **Freq** | **SCP** | **MCP** | **MCP_Ratio** | **TP** | **TP_rank** | **TC** | **TC_rank** | **Average Citations** |
| --- | --- | --- | --- | --- | --- | --- | --- | --- | --- | --- |
| CHINA | 75 | 0.286 | 62 | 13 | 0.173 | 311 | 1 | 1897 | 2 | 25.3 |
| USA | 44 | 0.168 | 25 | 19 | 0.432 | 235 | 2 | 2958 | 1 | 67.2 |
| JAPAN | 24 | 0.092 | 17 | 7 | 0.292 | 124 | 3 | 636 | 4 | 26.5 |
| UK | 18 | 0.069 | 10 | 8 | 0.444 | 54 | 6 | 951 | 3 | 52.8 |
| KOREA | 15 | 0.057 | 14 | 1 | 0.067 | 70 | 5 | 395 | 5 | 26.3 |
| SPAIN | 11 | 0.042 | 8 | 3 | 0.273 | 79 | 4 | 286 | 6 | 26 |
| SWEDEN | 11 | 0.042 | 5 | 6 | 0.545 | 50 | 7 | 232 | 8 | 21.1 |
| ITALY | 9 | 0.034 | 7 | 2 | 0.222 | 27 | 9 | 265 | 7 | 29.4 |
| GERMANY | 7 | 0.027 | 4 | 3 | 0.429 | 37 | 8 | 155 | 10 | 22.1 |
| INDIA | 6 | 0.023 | 5 | 1 | 0.167 | 21 | 12 | 149 | 11 | 24.8 |
| BRAZIL | 4 | 0.015 | 4 | 0 | 0 | 23 | 11 | 182 | 9 | 45.5 |
| NETHERLANDS | 4 | 0.015 | 0 | 4 | 1 | 13 | 14 | 80 | 15 | 20 |
| NORWAY | 4 | 0.015 | 0 | 4 | 1 | 10 | 17 | 85 | 14 | 21.2 |
| POLAND | 4 | 0.015 | 2 | 2 | 0.5 | 25 | 10 | 90 | 12 | 22.5 |
| CANADA | 3 | 0.011 | 1 | 2 | 0.667 | 20 | 13 | 29 | 17 | 9.7 |
| IRAN | 3 | 0.011 | 3 | 0 | 0 | 12 | 16 | 25 | 21 | 8.3 |
| AUSTRALIA | 2 | 0.008 | 1 | 1 | 0.5 | 6 | 23 | 5 | 25 | 2.5 |
| CHILE | 2 | 0.008 | 2 | 0 | 0 | 12 | 15 | 88 | 13 | 44 |
| AUSTRIA | 1 | 0.004 | 1 | 0 | 0 | 4 | 28 | 38 | 16 | 38 |
| DENMARK | 1 | 0.004 | 0 | 1 | 1 | 4 | 29 | 4 | 27 | 4 |

Note(s): Articles: Publications of Corresponding Authors only. Freq: Frequence of Total Publications. MCP_Ratio: Proportion of Multiple Country Publications. TP: Total Publications. TP_rank: Rank of Total Publications. TC: Total Citations. TC_rank: Rank of Total Citations. Average Citations: The average number of citations per publication.

**Table S6.** **Publication and citation profiles of leading countries in Scopus**

| **Country** | **Articles** | **Freq** | **SCP** | **MCP** | **MCP_Ratio** | **TP** | **TP_rank** | **TC** | **TC_rank** | **Average Citations** |
| --- | --- | --- | --- | --- | --- | --- | --- | --- | --- | --- |
| CHINA | 56 | 0.206 | 48 | 8 | 0.143 | 191 | 2 | 991 | 2 | 17.7 |
| USA | 48 | 0.176 | 31 | 17 | 0.354 | 305 | 1 | 3123 | 1 | 65.1 |
| JAPAN | 22 | 0.081 | 16 | 6 | 0.273 | 113 | 3 | 695 | 3 | 31.6 |
| KOREA | 14 | 0.051 | 12 | 2 | 0.143 | 44 | 5 | 483 | 4 | 34.5 |
| UK | 13 | 0.048 | 11 | 2 | 0.154 | 51 | 4 | 203 | 8 | 15.6 |
| ITALY | 10 | 0.037 | 8 | 2 | 0.2 | 33 | 10 | 334 | 5 | 33.4 |
| INDIA | 9 | 0.033 | 7 | 2 | 0.222 | 36 | 9 | 239 | 6 | 26.6 |
| SWEDEN | 7 | 0.026 | 4 | 3 | 0.429 | 30 | 11 | 139 | 9 | 19.9 |
| GERMANY | 6 | 0.022 | 3 | 3 | 0.5 | 37 | 8 | 110 | 10 | 18.3 |
| SPAIN | 6 | 0.022 | 3 | 3 | 0.5 | 40 | 6 | 61 | 15 | 10.2 |
| BRAZIL | 5 | 0.018 | 5 | 0 | 0 | 38 | 7 | 208 | 7 | 41.6 |
| CANADA | 5 | 0.018 | 3 | 2 | 0.4 | 25 | 12 | 67 | 13 | 13.4 |
| CHILE | 4 | 0.015 | 2 | 2 | 0.5 | 18 | 14 | 92 | 11 | 23 |
| MALAYSIA | 4 | 0.015 | 2 | 2 | 0.5 | 14 | 15 | 16 | 28 | 4 |
| NORWAY | 4 | 0.015 | 2 | 2 | 0.5 | 9 | 21 | 47 | 17 | 11.8 |
| POLAND | 4 | 0.015 | 2 | 2 | 0.5 | 19 | 13 | 92 | 12 | 23 |
| AUSTRALIA | 3 | 0.011 | 2 | 1 | 0.333 | 7 | 23 | 64 | 14 | 21.3 |
| FRANCE | 3 | 0.011 | 1 | 2 | 0.667 | 13 | 16 | 27 | 24 | 9 |
| AUSTRIA | 2 | 0.007 | 2 | 0 | 0 | 8 | 22 | 40 | 18 | 20 |
| IRAN | 2 | 0.007 | 1 | 1 | 0.5 | 10 | 17 | 29 | 20 | 14.5 |

Note(s): Articles: Publications of Corresponding Authors only. Freq: Frequence of Total Publications. MCP_Ratio: Proportion of Multiple Country Publications. TP: Total Publications. TP_rank: Rank of Total Publications. TC: Total Citations. TC_rank: Rank of Total Citations. Average Citations: The average number of citations per publication.

**Table S7**. **Publication and citation profiles of high-impact authors in WoSCC**

| **Author** | **h_index** | **g_index** | **m_index** | **PY_start** | **TP** | **TP_RANK** | **TP_FRAC** | **TC** | **TC_RANK** |
| --- | --- | --- | --- | --- | --- | --- | --- | --- | --- |
| WU Z | 9 | 9 | 0.692 | 2013 | 9 | 1.51 | 1 | 514 | 2 |
| NI JJ | 8 | 8 | 0.615 | 2013 | 8 | 1.01 | 3 | 449 | 3 |
| POTEMPA J | 8 | 9 | 0.571 | 2012 | 9 | 1.03 | 1 | 1402 | 1 |
| NAKANISHI H | 6 | 6 | 0.462 | 2013 | 6 | 1.5 | 6 | 365 | 4 |
| BLANCO J | 5 | 6 | 0.714 | 2019 | 6 | 0.7 | 6 | 104 | 15 |
| CHEN HW | 5 | 5 | 0.625 | 2018 | 5 | 0.57 | 8 | 342 | 7 |
| LIU YC | 5 | 5 | 0.385 | 2013 | 5 | 0.63 | 8 | 326 | 9 |
| SINGHRAO SK | 5 | 5 | 0.455 | 2015 | 5 | 1.64 | 8 | 329 | 8 |
| SONG ZC | 5 | 7 | 0.625 | 2018 | 7 | 0.78 | 4 | 350 | 5 |
| ZHOU W | 5 | 7 | 0.625 | 2018 | 7 | 0.78 | 4 | 350 | 5 |
| EICK S | 4 | 4 | 0.5 | 2018 | 4 | 0.45 | 14 | 84 | 19 |
| HE ZY | 4 | 4 | 0.8 | 2021 | 4 | 0.47 | 14 | 84 | 19 |
| KESAVALU L | 4 | 4 | 0.333 | 2014 | 4 | 0.5 | 14 | 198 | 12 |
| LEIRA Y | 4 | 5 | 0.571 | 2019 | 5 | 0.52 | 8 | 95 | 17 |
| LIAO Y | 4 | 4 | 0.667 | 2020 | 4 | 0.44 | 14 | 126 | 14 |
| QIAN XS | 4 | 5 | 0.667 | 2020 | 5 | 0.68 | 8 | 97 | 16 |
| QIU C | 4 | 5 | 0.667 | 2020 | 5 | 0.53 | 8 | 129 | 13 |
| SOBRINO T | 4 | 4 | 0.571 | 2019 | 4 | 0.35 | 14 | 92 | 18 |
| ZHANG X | 4 | 4 | 0.5 | 2018 | 4 | 0.44 | 14 | 316 | 10 |
| ZHOU YM | 4 | 4 | 0.308 | 2013 | 4 | 0.55 | 14 | 268 | 11 |

H index: The h-index of the journal, which measures both the productivity and citation impact of the publications. g index: The g-index of the journal, which gives more weight to highly-cited articles. m index: The m-index of the journal, which is the h-index divided by the number of years since the first published paper. TP: Total Publications. TP rank: Rank of Total Publications. TC: Total Citations. TC rank: Rank of Total Citations. Average Citations: The average number of citations per publication. PY start: Publication Year Start, indicating the year the journal started publication.

**Table S8**. **Publication and citation profiles of high-impact authors in Scopus**

| **Author** | **h_index** | **g-index** | **m-index** | **PY_start** | **TP** | **TP_Frac** | **TP_rank** | **TC** | **TC_rank** |
| --- | --- | --- | --- | --- | --- | --- | --- | --- | --- |
| WU Z | 8 | 8 | 0.889 | 2017 | 8 | 1.22 | 3 | 499 | 5 |
| NI J | 7 | 7 | 0.778 | 2017 | 7 | 0.89 | 7 | 486 | 6 |
| KAMER AR | 6 | 7 | 0.333 | 2008 | 7 | 0.7 | 7 | 869 | 2 |
| POTEMPA J | 6 | 7 | 0.75 | 2018 | 7 | 0.76 | 7 | 1396 | 1 |
| ZHANG Y | 6 | 10 | 0.857 | 2019 | 10 | 1.37 | 1 | 214 | 9 |
| DE LEON MJ | 5 | 5 | 0.278 | 2008 | 5 | 0.48 | 10 | 809 | 3 |
| LI Y | 5 | 9 | 0.833 | 2020 | 9 | 1.28 | 2 | 142 | 11 |
| LIU X | 5 | 8 | 0.833 | 2020 | 8 | 1.14 | 3 | 169 | 10 |
| LIU Y | 5 | 8 | 0.556 | 2017 | 8 | 1.03 | 3 | 343 | 7 |
| SINGHRAO SK | 5 | 8 | 0.417 | 2014 | 8 | 2.39 | 3 | 287 | 8 |
| CRAIG RG | 4 | 4 | 0.222 | 2008 | 4 | 0.4 | 11 | 777 | 4 |
| EICK S | 4 | 4 | 0.5 | 2018 | 4 | 0.45 | 11 | 87 | 16 |
| OLSEN I | 4 | 4 | 0.5 | 2018 | 4 | 3.1 | 11 | 73 | 17 |
| WANG Z | 4 | 4 | 0.667 | 2020 | 4 | 0.54 | 11 | 52 | 18 |
| YANG H | 4 | 4 | 0.308 | 2013 | 4 | 0.42 | 11 | 108 | 14 |
| CARRERAS I | 3 | 3 | 0.5 | 2020 | 3 | 0.33 | 17 | 119 | 12 |
| CHEN H | 3 | 3 | 0.6 | 2021 | 3 | 0.38 | 17 | 88 | 15 |
| CHENG X | 3 | 4 | 0.75 | 2022 | 4 | 0.62 | 11 | 38 | 19 |
| DEDEOGLU A | 3 | 3 | 0.5 | 2020 | 3 | 0.33 | 17 | 119 | 12 |
| GAO C | 3 | 3 | 1 | 2023 | 3 | 0.38 | 17 | 35 | 20 |

H index: The h-index of the journal, which measures both the productivity and citation impact of the publications. g index: The g-index of the journal, which gives more weight to highly-cited articles. m index: The m-index of the journal, which is the h-index divided by the number of years since the first published paper. TP: Total Publications. TP rank: Rank of Total Publications. TC: Total Citations. TC rank: Rank of Total Citations. Average Citations: The average number of citations per publication. PY start: Publication Year Start, indicating the year the journal started publication.

**Table S9**. **Keyword occurrences analysis.**

| **WoSCC** | | | **Scopus** | |
| --- | --- | --- | --- | --- |
| **Keywords** | **Occurrences** | **Total link strength** | **Keywords** | **Occurrences** |
| alzheimers-disease | 91 | 331 | alzheimer disease | 311 |
| dementia | 56 | 245 | periodontitis | 273 |
| inflammation | 50 | 224 | human | 206 |
| tooth loss | 42 | 195 | article | 183 |
| porphyromonas-gingivalis | 37 | 167 | porphyromonas gingivalis | 177 |
| association | 38 | 162 | female | 151 |
| brain | 37 | 149 | humans | 150 |
| oral-health | 29 | 134 | male | 142 |
| periodontitis | 40 | 129 | aged | 131 |
| health | 34 | 126 | controlled study | 101 |
| risk | 32 | 119 | nonhuman | 96 |
| expression | 25 | 82 | inflammation | 83 |
| periodontal-disease | 20 | 77 | middle aged | 75 |
| disease | 21 | 75 | periodontal disease | 75 |
| prevalence | 16 | 73 | adult | 72 |
| risk-factor | 14 | 65 | metabolism | 70 |
| activation | 16 | 62 | animal | 69 |
| decline | 12 | 62 | mice | 63 |
| infection | 11 | 59 | cognitive defect | 57 |
| cognitive impairment | 12 | 55 | mouse | 53 |
